# Fast Prototyping Ceramic Gas Flow Sensors for Harsh Operating Conditions

**DOI:** 10.3390/mi17020188

**Published:** 2026-01-30

**Authors:** Andrey Kasenko, Pavel Shchur, Ekaterina Anatolevna Drach, Ivan Borzunov, Vasily V. Egorov, Boris Prudnikov, Konstantin Oblov, Arthur Litvinov, Yuri Voronov, Nikolay Samotaev

**Affiliations:** 1 INTER Limited Liability Company, 115201 Moscow, Russia; kasenko@roost95.ru (A.K.); shur-pavel@mail.ru (P.S.); catherinedratch@yandex.ru (E.A.D.); vanyaborzunov@yandex.ru (I.B.); vsem_dobra@bk.ru (V.V.E.); prudnikov.borya@yandex.ru (B.P.); 2 Micro- and Nanoelectronics Department, National Research Nuclear University MEPhI (Moscow Engineering Physics Institute), 115409 Moscow, Russia; kyoblov@mephi.ru (K.O.); avlitvinov@mephi.ru (A.L.); yavoronov@mephi.ru (Y.V.)

**Keywords:** gas flow sensor, ceramics, laser micromachining, fast fabrication

## Abstract

The technology development for the mass ceramic gas flow sensor (CGFS) adopted for harsh operating conditions is presented. The main characteristic of this technology is its simplicity and affordability for mass fast prototyping of CGFS with a limited set of technological equipment. Special attention is paid to the discussion of the technological and operational materials’ compatibility, flexibility, and speed of their processing to adapt the best mass flow sensor design option. The CGFS, designed and manufactured in just a few days, was tested in conditions close to the real ones and demonstrated the ability to measure gas flow in the range from 0.21 m/s to 1.25 m/s, with a constant power consumption of 152 mW@346 °C.

## 1. Introduction

There has always been great interest in ceramic gas flow sensor (CGFS) in the microelectromechanical systems (MEMS) commercial market since the first micromachined thermal flow sensor fabrication in 1974 [1]. Gas flow sensors are currently widely used in various fields, from medicine [2] and domestic public utilities [3] to car combustible engines [4], etc. All commercial gas flow sensors are fabricated using microelectronic technology, and they can be classified by the technology they are manufactured with. In the vast majority, there are only three cases: silicon [5], glass [6], and ceramic [7]. Micromachined flow sensors can be classified by the physical principle of work as either thermal or non-thermal. The different constructions of MEMS flow sensors are more precisely discussed in review publications [8,9]. In this work, we focus on thermal ceramic gas flow sensors, which are widespread on the commercial market and are well studied. The simple design of thermal CGFS without moving parts simplifies design rules and fabrication requirements. Another reason for a lot of interest in thermal flow sensors is the advantages gained through MEMS miniaturization: low power consumption, higher sensitivity to low flow rates, and easy use in different modes of operation (pulse and constant heating). Thermal gas flow sensors during his work must be heated in the open air in unpredictable conditions or in an atmosphere with aggressive components, which may generate a chemical reaction. For these reasons, we can say that gas flow sensors (unless they are strictly specialized for a specific application) always operate under harsh (severe) operating conditions compared to standard integrated circuits’ (ICs’) specifications (microcontrollers, amplifiers, frequency filters, etc.), which are always sealed in a package from the external atmosphere using inert nitrogen gas or plastic. The example of harsh operating conditions is the simplest medical application of a gas flow sensor in a breath test [10], which has the risk of condensation of the moisture (saliva) exhaled by a person on the sensitive elements’ surface (micro heater, thermistors). Based on the electronic materials classification and technologies presented in the review publication [11], we can say that ceramic microelectronic devices manufactured using post-fired technology have the greatest resistance to harsh operating conditions. Therefore, the main task is to select the right materials and sensor technology concepts that will allow us to realize the target for our fabrication of a fully functional prototype CGFS for harsh environmental conditions (high temperature and aggressive chemical components) in just a few days.

## 2. Concept of Sensor Prototyping Technology

There are ZrO_2_ type 3YSZ wafers (tetragonal zirconia oxide stabilized with the addition of 3 mol% Y_2_O_3_ [12]), which boast the best mechanical and electrical properties for all commercially available modifications. They were selected as the material for easy use and for the best isolation resistance to harsh operating conditions. The use of this type of 3YSZ as oxygen and nitrogen oxide sensors in diesel engine exhaust gases demonstrates the material’s excellent long-term stability under the harshest gas flow conditions, temperature, and vibration loads [13]. The initial easy use and robustness (reliability) of the flow sensor design are due to the many physical properties of the ceramic wafer and its platinum metallization, which guarantees high mechanical strength during long-term thermal cycling. The materials’ coefficients of thermal expansion are virtually identical—10.5 × 10^−6^/K and 9.4 × 10^−6^/K, respectively. This value is one of the lowest among all metals, making platinum a highly stable material when exposed to temperature fluctuations. The matching of thermal expansion coefficients is not only an important operational aspect, but also a technological advantage due to the availability of thin membrane ZrO_2_ (up to 20 µm) fabricated by type casting [14].

The advantage in the tech aspect for ZrO_2_ lies in the ability to cover the surface with a continuous layer and deposit a relatively thick platinum layer (over 1 µm) without the use of specialized shadow mask-like equipment needed to reduce emerging mechanical stress in the membrane. A good illustration of this advantage is the authors’ attempt to vacuum-deposit an initial continuous 1 µm platinum layer onto 30 µm-thick SCHOTT AF 32^®^ eco thin glass [15]. While still in the vacuum chamber during platinum deposition (upon reaching the required metal thickness), the glass began to roll up the substrate until the substrate itself cracked under mechanical stress. An additional advantage of using platinum is its chemical inertness and resistance to interaction with acidic environments (except for boiling in aqua regia). For the electrical circuit for reading the signal (more details in the next section), platinum is the best material, having the highest temperature coefficient of resistance among metals of 0.003927 and a low thermal conductivity of 71.6 W/(m × K).

Having identified the best gas flow sensor materials, it is necessary to determine the optimal manufacturing strategy (technology) that will quickly yield the best possible topology. Recognizing that experiment is a key criterion for validity, as it allows theories and hypotheses to be empirically tested in real-world conditions, confirming or refuting them through observation, repeatability, and obtaining reliable data, laser micromachining can be chosen as the technology of choice. Laser micromachining offers the following advantages:

- The chip’s topology and size can be changed literally in-line;
- Chip fabrication speed is 1–2 min, i.e., a negligible amount of time compared to other microelectronic technologies (photolithography, chemical, or ion etching, etc.)
- In a single cycle, both the metallization topology and the chip contour are manufactured from the substrate, meaning that even within a single chip, we have a high degree (sensitive element precision positioning on the substrate) of positioning of the sensing element relative to the substrate.
- The chip holder (package) is manufactured on the same equipment, ensuring compatibility of system sizes made from different materials (ceramics);
- The cost of equipment for the technological process is minimal (laser marker machine);
- No cleanroom is required for the technological process.

An important aspect beyond sensor manufacturing is its initial long-term stability testing. Since time is the primary non-renewable resource in our work, it is best to combine accelerated testing with the sensor manufacturing process. Unfortunately, laser micromachining is a “dirty” process, leaving behind a large amount of nanoparticle dust after ablation, which must be cleaned in an ultrasonic bath. A criterion for good long-term stability of the platinum metallization topology can be its resistance to ultrasonic treatment, especially after high-temperature annealing to stabilize resistance (more details in Section 3). Of course, rejecting chips at the manufacturing stage does not negate testing on a specialized gas flow measurement stand; it merely provides additional confidence in the quality of the manufactured sensors. To summarize the above points, it can be concluded that with a minimal set of equipment—a laser marker, a digital video camera, an ultrasonic bath, a muffle furnace, and a specialized gas flow measurement stand (either self-made or purchased commercially [16])—it is possible to manufacture a fully functional gas flow sensor suitable for heavy-duty operating conditions.

## 3. Sensor Realization and Integration

**Figure 1:**
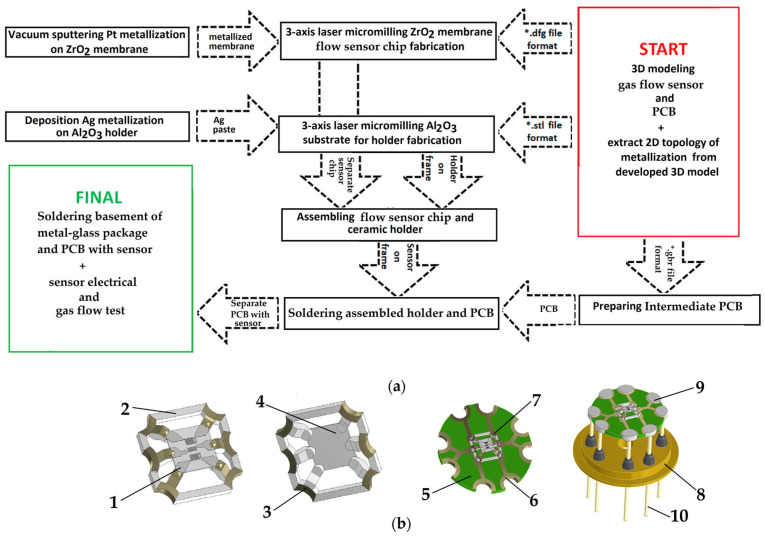
Technological steps used for flow sensors fabrication: (**a**) full flowchart for sensors fabrication; (**b**) 3D model of flow gas sensor in the TO-8 package: 1—Pt metallization; 2—Al_2_O_3_ ceramics; 3—Ag metallization; 4—ZrO_2_ membrane; 5—printed circuit board (PCB) for holder mounting; 6—Au contact pads for soldering pins of metal-glass package; 7—Sn/Pb solder; 8—TO-8 package basement; 9—Sn/Pb solder; 10—pins of metal-glass package for flow sensors electrical testing. "*" means any value of the file name.

To fabricate ceramic parts of the CGFS, we used a digital technological flow (as presented in Figure 1a). We developed a 3D model of the sensing device by using 3D modeling software (COMPAS-3D Home (Russian Federation) [17]). As a result, the file was in STL format (as presented in Figure 1b). The special 20 W fiber laser with tunable pulse duration in the range of 50–200 ns and a wavelength of 1.064 µm, controlled by specially produced software [18], was used to fabricate different parts of the developed sensor. The size of the focused laser spot during micro processing was 25 µm (a lens with a field of view of 50 × 50 mm was used). This approach allowed us to combine the process of micromilling with a digital comparison of the fabricated devices, their geometrical parameters within a 3D model, and the achieved quality after the fabrication process. Additionally, from the 3D model, an extraction of the chip metallization in a DFG file format and GRB file (GERBER) format for the manufacture of an industrial printed circuit board (PCB) was made. DFG files are needed for the acceleration of searching for the best topology metallization because the flow sensor chip design is planar and does not require 3D laser micromilling.

**Figure 2:**
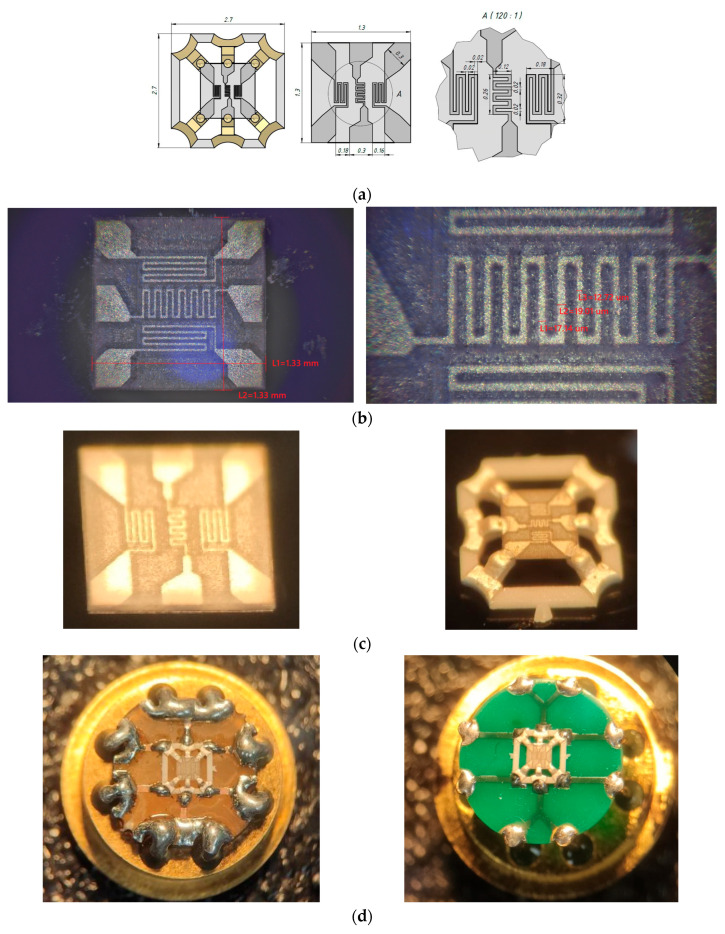
Dimension of gas flow sensor: (**a**) sketch and topology dimensions of the membrane part of gas flow sensor; (**b**) measuring the resulting membrane (**left**) and platinum micro heater parts of gas flow sensor using digital microscope after laser processing; (**c**) optical photo of ZrO_2_ membrane with gas flow sensor micro heater (**left**) mounted on ceramic holder (**right**); (**d**) optical photo of soldered ceramic holder with flow sensor chip onto hand-made PCB (**left**) and industrial-made PCB (**right**) soldered on the TO-8 metal-glass package.

Throughout the fabrication process that took place in an air atmosphere, the laser parameters were tuned to satisfy the specific requirements of each step, as listed in Table 1. The ceramic dust debris (present in Figure 2b, left image) removal methods after laser processing were ultrasonic cleaning.

On a thin, 30 micron-thick ceramic ZrO_2_ membrane with dimensions 1.3 × 1.3 mm fabricated by the 3YSZ material type, a membrane thin-film platinum heater in the form of a meander with a resistance of 24 ohms, and two thermistors were formed by laser micromilling. The topology of the micro heater is shown in Figure 2. Platinum metallization with a thickness of 1 µm was deposited on a ZrO_2_ membrane by magnetron sputtering and annealed for several hours in a kiln with an air atmosphere at 950 °C to stabilize the resistance of the micro heater by a physical mechanism, as described in [19]. During annealing, the micro heater resistance decreased several times, and the signal to stop annealing was when it ceased to change by more than 1%. The resulting chip with micro heater elements was mounted on a ceramic holder made of monolithic 96% alumina by two-sided laser micromilling [20], the photo of which is shown in Figure 2d. The metallization of the ceramic holder is based on thick-film silver doped by Pt paste (type PP-33 [21]) and has good solderability properties with standard solders (we used 60% Sn/40% Pb solid solder). The ceramic holder with a mounted flow sensor chip based on the ZrO_2_ membrane was soldered onto an intermediate printed circuit board, and after that, the entire construction was mounted on an 8-outlet metal-glass package TO-8 standard using pressure sensors [22].

**Figure 3:**
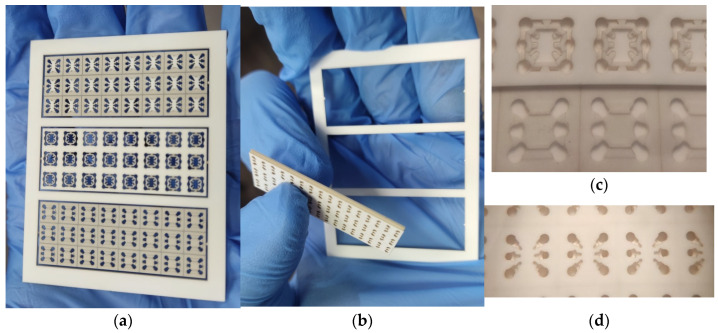
Photo of ceramic mask for depositing thick-film metallization to the alumina ceramic holder: (**a**) photo of fabricated two part of mask and ceramic holder on one substrate; (**b**) photo of all three parts removed from the alumina substrate and their easy gap-free alignment; (**c**) photo of the location of the holder inside the mask; (**d**) closed mask before Ag metallization deposition (the top view in optical microscope).

One of the key features for safe time in this work was the use of a three-dimensional ceramic mask to deposit thick-film metallization on the ceramic holder. Two parts of the mask (upper and bottom) were manufactured by laser micromilling in a single cycle with the ceramic holder on the same 60 × 48 × 0.5 mm size substrate. This approach avoided dimensional misalignment when aligning the mask parts and the holder chips. The single 96% alumina substrate contained 3 × 8 = 24 ceramic holders, which is presented in Figure 3. The substrate with holders and masks was fabricated over 8 h on the first day of the experiment, in parallel to the vacuum deposition of Pt metallization. Another small innovation that accelerated the production and testing of sensor chips was the placement of ceramic holders in the substrate on jumpers (jumper size is a pyramid with a top size of 100 × 100 µm). Chips with membrane sensor elements were laid out on holders and fired once in a group onto silver paste. After the firing process, we conducted electrical testing and selected a suitable (by resistance of micro heater) flow sensor already based on the ceramic holder without destroying the entire frame of workpiece—this is very convenient for subsequent use, when the topology of the sensor element changes many times, but the shape of the holder remains the same. The use of silver paste adopted for the post-firing tech process allows for multiple re-firing in air at 850 °C. The firing condition 850 °C (15 min) will not change anything (ohmic resistance) for already stabilized platinum metallization.

All experimental work on manufacturing the sensor took several days (the first day vacuum sputtering of Pt metallization and annealing, and the second day laser micromilling and mounting the sensor chip on a ceramic holder), including two days of waiting for the fast production of a PCB in factory conditions. A PCB is needed to check the soldering possibilities of the ceramic holder to the standard, most widespread industrial ENIG (electroless nickel immersion gold) finish coating. Of course, we tried to reduce the development time and immediately confirm the ceramic holder’s soldering capability. Therefore, in parallel with ordering an industrial PCB, we fabricated by laser micromilling our own PCB for a TO-8 package within a few minutes, unfortunately, without the necessary final coatings, but which passed the soldering test. The image of the final sensor with various PCBs (hand-made and industrial) provided for flow measuring tests is shown in Figure 2c.

## 4. Experimental Results and Discussion

**Figure 4:**
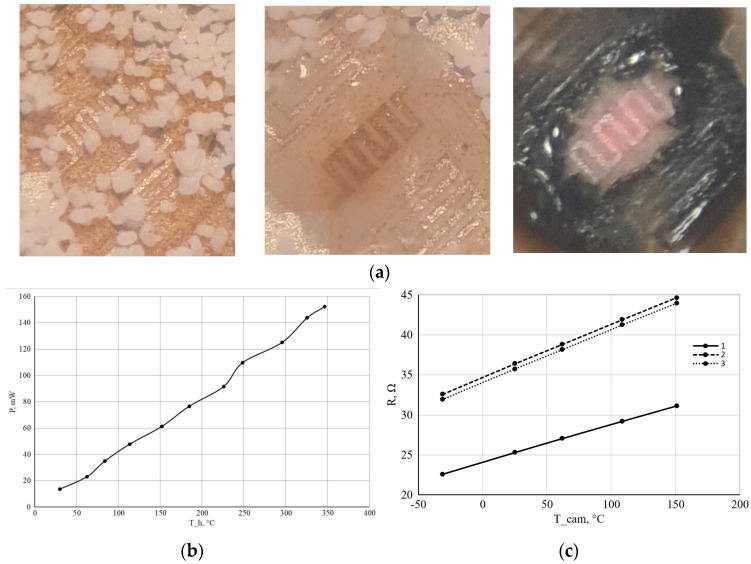
Experiment to determine the thermal coefficient of resistance and power consumption: (**a**) platinum micro heater on ZrO_2_ membranes under melting point technique evaluation (from left to right, the temperature increases until the polyamide powders species melts (**center**) and burns (**right**); (**b**) the dependence of the temperature on the power consumption for micro heater element; (**c**) dependence of the resistance on the ambient temperature for the heater (R_h_, curve 1) and thermistors (R_1_, curve 2 and R_2_, curve 3).

Sputtered Pt-heaters with an appropriate resistance in a range of 24 ohms were tested by a micro-melting technique [23] at temperatures ranging up to 600 °C. To conduct these tests, we used a 50 µm grain size polyamide micro powder [24] with a melting point of 180 °C to understand the dependence of power consumption on working temperature at the surface of a micro heater, which is presented in Figure 4c. The platinum metallization on the ZrO_2_ membrane has 0.00197 1/°C temperature coefficient of resistance (TCR). The temperature-power consumption relationship of the micro heater is presented in Figure 4. Power consumption was calculated using applied voltage and current. The power consumption testing shows 125 ± 5 mW for a 300 °C working temperature from sample to sample, fabricated on the same membrane (see Figure 4). Using the micro-melting technique is a crude method, but it helps visualize the dynamics of thermal wave propagation on the sensor surface. This is especially evident when the powder has already melted on the micro heater but has not yet melted on the thermistors.

**Figure 5:**
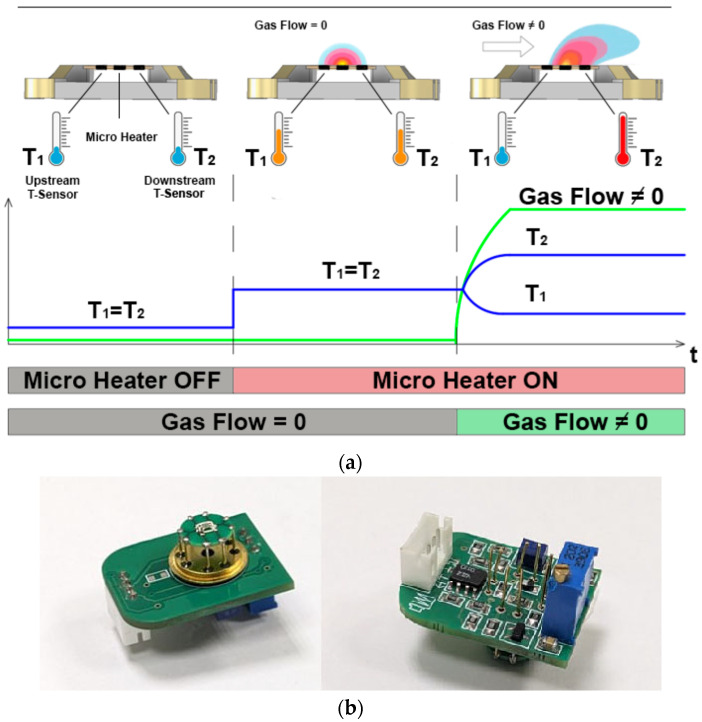

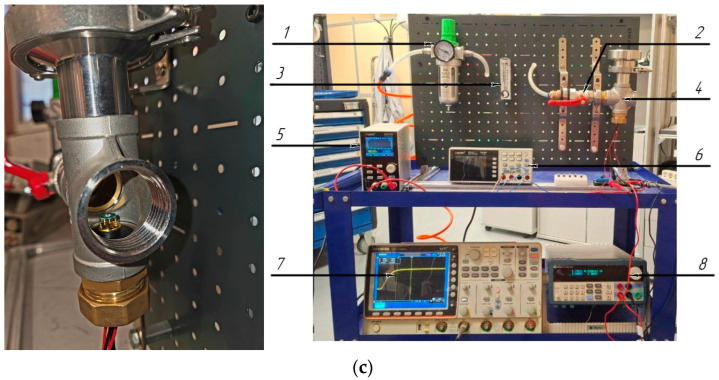
Experiment to determine gas flow: (**a**) typical gas flow measurement experiment scheme with two thermistors and one micro heater; (**b**) photo of electronic control module from one side of PCB (**right**) with fabricated gas flow sensor (**left**) and on another side of PCB used for experiments; (**c**) the photo of experimental setup on which characteristics of the developed sensor are measured (**right**) and photo of sensor integration in tube with changeable gas flow (**left**). The decoding of the numbers under which the measurement devices are indicated in the photo is given in the text.

Having determined the temperature coefficient of resistance, it is possible to continue testing, for which the sensor was selected (Figure 5b). The resistance of the heater (R_H_) and thermistors (R1 and R2) at 25 °C is 25.71 ohms, 36.37 ohms, and 35.73 ohms, respectively (for sensor #2). It is important to note that the difference in thermistor resistances is less than 2%, which can be considered a good result for this technology. Since the temperature difference between the thermistors (R_1_ and R_2_) must be known to perform the measurements, the TCR measurements were performed by another method using a climate chamber KTH-74 [25] with a generated temperature range of −65 to +165 °C. The obtained values are shown in Figure 4. The figure shows that the resistances are linear with temperature. The TCR value can be determined using the following formula:

(1) R_0_ = R (1 + α (T_0_ − T)),

where R_0_ and T_0_ are the initial resistance and temperature (25 °C), and R is the resistance at temperature T. The average values of the TCR of the heater (R_h_) and thermistors (R_1_ and R_2_) for all temperatures were 0.00184 1/°C, 0.001836 1/°C, and 0.001864 1/°C, respectively. These results are in good agreement with the micro-melting experiment conducted previously. After the second experiment to determine the TCR, the results of which were taken as the main ones, an experiment was already carried out using the scheme, as shown in Figure 5a.

To measure the flow sensor response depending on the mass flow rate of gas, the experimental setup was assembled (Figure 5c), including a steel section of gas pipe (32 mm in diameter), in which the sensor was located {4}, a gas flow control valve {1}, a mechanical gas rotameter {3}, a hand shutter {2} and a compressor {not present on photo}, as well as a power source {5}, a special waveform generator {6}, a milliohmmeter and millivoltmeter {8} and an oscilloscope {7}, for heating the sensor to operating temperature and taking electrical measurements. The measured parameters are gas flow and temperature (resistance of the thermistors). A photo of the electronic control module designed for the gas flow sensor used for experiments is presented in Figure 5b.

The developed electronic control module controls the heater heating, as well as amplifies, corrects, and switches the potential across the sensor’s thermistors. The electrical circuit implements two measurement modes: inlet temperature measurement and gas mass flow measurement. To measure gas mass flow, the thermistors are connected to a bridge measuring circuit. The gas mass flow measurement cycle consists of two stages. During the first stage, the heater is heated to the required temperature. During the second stage, the response from the bridge circuit is measured. The heating current is controlled using a pulse-width modulation (PWM) signal. To measure the sensor’s dynamic characteristics, it was connected to a divider circuit (R_d_ = 10 Ohm). The sensor heater was heated to operating temperature using a PWM signal with a frequency of 1 kHz and a fill factor corresponding to the required operating voltage. The 3.7 V lithium battery served as the power source. The PWM was generated by applying rectangular pulses to the gate of a field-effect transistor from a special waveform generator. During this process, the heater R_H_ heated up, causing its resistance to increase. This resulted in a change in voltage across the divider’s resistor Rd, which was measured with an oscilloscope.

**Figure 6:**
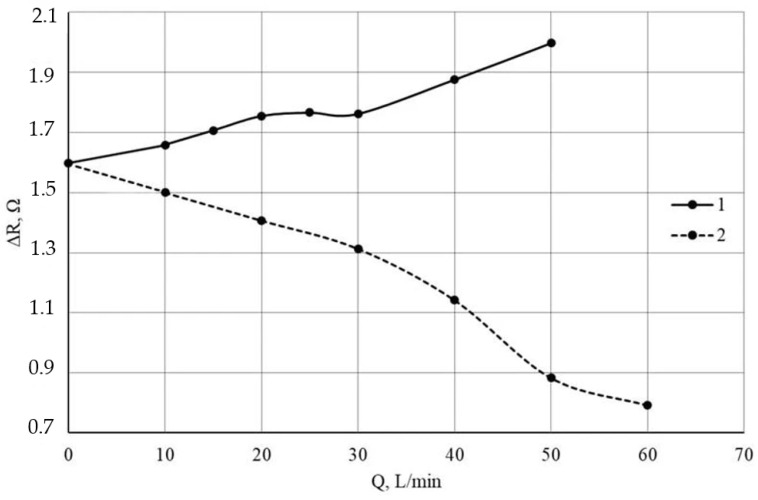
Dependence of _∆_R on the gas flow rate when R_1_ is behind the heater (1) and in front of the heater (2) (i.e., the flow is moving in opposite directions).

Using Formula (1), the micro heater and thermistor temperatures were calculated as functions of the voltage applied to the micro heater. From the obtained dependences, it can be concluded that the maximum heater temperature (T_n_) at a voltage of 2.5 V is 346.8 °C. The power consumed by the heater, P_n_, is 152.3 mW. The temperatures of the thermistors (T_1_ and T_2_) in the absence of gas flow are practically equal, 220.1 °C and 227.8 °C, respectively. After determining the temperature (resistance) under no-flow conditions, an experiment was conducted with air flow rates in the range from 10 m/min to 50 L/min, the results of which are presented in Figure 6.

The measurements were carried out under laminar gas flow conditions. In the future, it is assumed that the sensor will be placed in the microchannel of the bypass outlet from the main gas pipe, and the laminar flow condition will be observed in all modes. This solution is typical for all mass production devices of this class.

Figure 6 shows the change in the resistance difference of the thermistors _∆_R = R_1_ − R_2_ depending on the air flow rate (Q) at a heater voltage of 2.4 V. It is important to note that the change in _∆_R depends on the sensor’s position in the gas flow. Dependences 1 and 2 in Figure 6 are the output signals of the thermistors of the same sensor when the flow is moving in opposite directions. When rotated 180 degrees, an increase in _∆_R is replaced by a decrease in _∆_R relative to the gas flow rate. This behavior of the resistance difference _∆_R is a consequence of the unevenness of their initial heating in the absence of gas flow. Thermistor R_1_ has a higher temperature than thermistor R_2_ (Figure 4c). In this case, when sensor R_1_ is located behind the heater, the difference _∆_R will increase due to the fact that sensor R_2_ will cool and sensor R_1_ will additionally heat up. When R1 is located in front of the heater, its temperature, and consequently _∆_R, will decrease. Figure 6 shows the _∆_R = f(Q) dependences, as these are more informative for explaining the obtained results than the dependences of the U_out_ response of the bridge circuit on Q. When a voltage is applied to the bridge circuit, ensuring a current of 1 mA through the thermistors, the voltage U_out_ will vary in the range from 0.8 mV to 2 mV. The value of Q is interesting from a gas flow rate control perspective. From a physical perspective, the gas flow rate V is a more useful quantity. The gas flow rate is determined by the following equation:

(2) V = Q/S,

where Q is the gas flow rate in m^3^/s, and S is the cross-sectional area of the pipe in the experiment. In our case, the pipe diameter is 32 mm. Then, S = 8 × 10^−4^ m^2^. The change in flow rate from 10 L/min to 60 L/min corresponds to a change in Q from 1.67 × 10^−4^ m^3^/s to 1 × 10^−3^ m^3^/s. Therefore, using Formula (2), when the flow rate changes from 10 L/min to 60 L/min, V will change from 0.21 m/s to 1.25 m/s.

**Figure 7:**
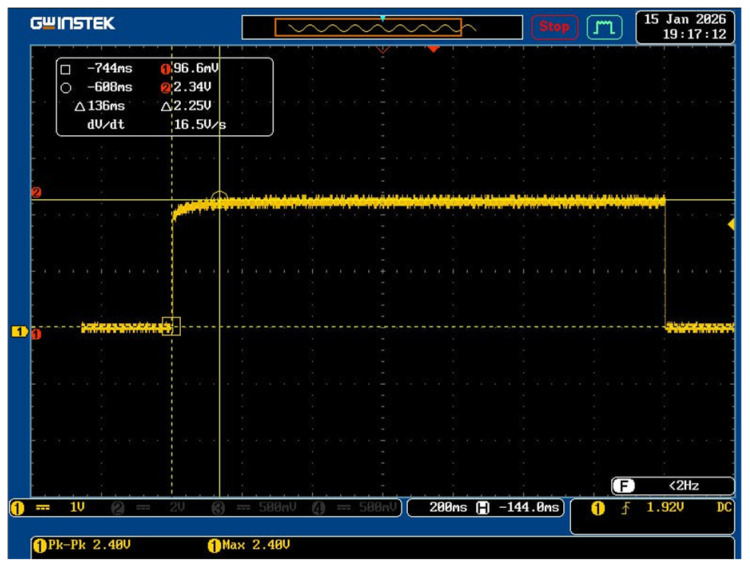
Dynamic dependence of the sensor output time on the operating mode.

Measurements of the volume of gas flowing in the pipeline through the section are usually carried out in a periodic mode. Therefore, the time for the sensor to enter the operating mode is important, which corresponds to heating the sensor to the set temperature. Figure 7 shows the dependence of the voltage change on the heater on the time of application of the operating voltage. To carry out measurements, the sensor heater was connected to a circuit with a voltage divider. It can be seen that when an operating voltage of 2.4 V is applied, the sensor heater heats up in 136 ms.

The sensor is designed to detect the flow of flammable gases in residential areas. In particular, the sensor must operate at temperatures of the measured medium from −25 °C to +55 °C. Nevertheless, the materials used in the sensor are capable of operating in harsh conditions. The ultimate goal of the research is to create a sensor capable of working for a long time (more than 6 years) from an autonomous power source (lithium battery, type D). To do this, it is necessary to ensure the minimum energy consumption of the sensor during a single measurement. This, in turn, requires minimizing the power consumption in the measurement mode and the time for the sensor to enter the operating mode. Both of these parameters are supposed to be reduced by further miniaturization of the sensor. In addition, energy consumption can be further reduced by changing the measurement accuracy. Through optimization, it is expected to reduce the average power consumed by the sensor to less than 1 mW when measured once per minute.

## 5. Conclusions

The miniature MEMS bulk CGFS was designed and manufactured by laser micromachining. The sensor’s design differs from the classic CGFS, which are resistors deposited on a 200-µm-thick alumina flat plate [7]. Our CGFS’s zirconia membrane is 30 µm thick, which is almost an order of magnitude thinner. Our sensor’s bulk design is more similar to silicon MEMS sensors, but is made of ceramic. The use of ceramics as a construction material allows us to claim that the sensor can be used in harsh conditions and at high temperatures (up to 450 °C and more), which would not be possible for experimental gas flow sensors produced by technology close to that described in our work—i.e., single-step laser patterning of different metals on organic substrates [26,27,28]. The developed sensor’s thermistors have low resistance compared with classical commercial thermistors of thermal flow sensors (typical value is 1K [5,6,7]) and give a chance to use them as a heating element, also to prevent condensation on the membrane surface. Figure 4a shows the possibility of burning polyamide powder and the subsequent soot during overheating of the micro heater—phenomena that are impossible to see on the standard membrane of the silicone gas flow sensors. All this allows us to assert the possibility of overcoming various scenarios (condensation of liquids, carbon-containing particles, etc.) when using the developed sensor in harsh conditions. The experimental, obtained resolution values of gas flow indicate that CGFS is fully functional. Power consumption at operating temperature is relatively high for use in autonomous, battery-powered devices in constant heating mode, but it is possible in pulse heating with a low duty cycle. By using higher-quality laser equipment (for example, with a 5-µm laser spot instead of the 25-µm spot used in our work), power consumption can be halved due to greater miniaturization of the metallization topology. Moreover, further sensor chip miniaturization is possible without changing the technological approaches described in this article. The developed construction of CGFS may have low-scale production due to the use of advanced microelectronic technologies compared to the classic one. The main advantage of the described technology is the time and cost reduction for low–medium scale production. Saving time and cost is achieved by not requiring the preparation of tooling (photo masks, printing stencils, vacuum deposition tooling like shadow masks, etc.) to produce the CGFS.

## Figures and Tables

**Table 1:** Laser parameters for the steps of the fabrication process.

Process/Material	Irradiation Times [-]	Power [*W*]	Speed [*mm s*^−1^]	Freq. [*Hz*]	Pulse Duration [*ns*]
Ablation metallization, Pt	3	0.6	100	20	100
Sensor chip cutting, ZrO_2_	100	1	200	20	100
Layer-by-layer milling of ceramics, Al_2_O_3_	63	18	300	20	200

## Data Availability

The original contributions presented in this study are included in the article.

## References

[1] Vanputte A.F., Middelho S. (1974). Integrated silicon anemometer. Electron. Lett..

[2] Silvestri S., Schena E. (2012). Micromachined Flow Sensors in Biomedical Applications. Micromachines.

[3] Ficco G., Dell’Isola M., Graditi G., Monteleone G., Gislon P., Kulaga P., Jaworski J. (2024). Reliability of Domestic Gas Flow Sensors with Hydrogen Admixtures. Sensors.

[4] Hourdakis E., Sarafis P., Nassiopoulou A.G. (2012). Novel Flow Meter for an Automobile Engine Using a Si Sensor with Porous Si Thermal Isolation. Sensors.

[5] Innovative Sensor Technology IST AG Silicon Flow Sensor SFS01. https://www.ist-ag.com/en/products/silicon-flow-sensor-sfs01.

[6] Innovative Sensor Technology IST AG MicroFlowSens MFS02 Thermal Gas Flow Sensor. https://www.ist-ag.com/en/products/microflowsens-mfs02-thermal-gas-flow-sensor.

[7] Innovative Sensor Technology IST AG Thermal Gas Flow Sensor FS7. https://www.ist-ag.com/en/products/thermal-gas-flow-sensor-fs7.

[8] Kuo J.T.W., Yu L., Meng E. (2012). Micromachined Thermal Flow Sensors—A Review. Micromachines.

[9] Balakrishnan V., Phan H.-P., Dinh T., Dao D.V., Nguyen N.-T. (2017). Thermal Flow Sensors for Harsh Environments. Sensors.

[10] Hedrich F., Kliche K., Storz M., Billat S., Ashauer M., Zengerle R. (2009). Thermal flow sensors for MEMS spirometric devices. Procedia Chem..

[11] Pradhan D.K., Moore D.C., Francis A.M., Kupernik J., Kennedy W.J., Glavin N.R., Olsson R.H., Jariwala D. (2024). Materials for high-temperature digital electronics. Nat. Rev. Mater..

[12] American Elements 3% Yttria Stabilized Zirconia. https://www.americanelements.com/3-yttria-stabilized-zirconia-113482-02-3.

[13] Wang J., Li X., Wang Z., Feng J., Lin W., Peng J. (2022). Research on Application Characteristics of Zirconia-Based High-Temperature NOx Sensors. Energies.

[14] Kyoritsu Elex Co., Ltd Zirconia Ceramic Substrate. https://www.kyoritsu-po.co.jp/products/zirconia-ceramic-substrates/.

[15] The SCHOTT Group AF 32^®^ Eco. https://www.schott.com/en-gb/products/af-32-eco-p1000308.

[16] Sensirion A.G. Gas Flow Sensors Evaluation. https://sensirion.com/products/sensor-evaluation/gas-flow-sensors-evaluation.

[17] COMPAS-3D Home. https://kompas.ru/kompas-3d-home/about/.

[18] Samotaev N., Oblov K., Ivanova A., Gorshkova A., Podlepetsky B. Rapid Prototyping of MOX Gas Sensors in Form-Factor of SMD Packages. Proceedings of the 2019 IEEE 31st International Conference on Microelectronics (MIEL).

[19] Kalinin I.A., Roslyakov I.V., Tsymbarenko D.M., Bograchev D.A., Krivetskiy V.V., Napolskii K.S. (2021). Micro heaters based on Pt and Pt-Rh films: The impact of composition, structure, and thermal treatment on functional properties. Sens. Actuators A Phys..

[20] Samotaev N., Oblov K., Dzhumaev P., Fritsch M., Mosch S., Vinnichenko M., Trofimenko N., Baumgärtner C., Fuchs F.-M., Wissmeier L. (2021). Combination of Ceramic Laser Micromachining and Printed Technology as a Way for Rapid Prototyping Semiconductor Gas Sensors. Micromachines.

[21] Semiconductor Ag-Pd-Pt Pastes. https://elma-paste.ru/paste_pt/.

[22] TO-8 Style Pressure Sensor Package. https://z-mars.ru/produktsiya/korpusa-datchikov/korpusa-metallosteklyannye-33018-to-8.html.

[23] Biró F., Dücso C., Hajnal Z., Riesz F., Pap A.E., Bársony I. (2015). Thermo-mechanical design and characterization of low dissipation micro-hotplates operated above 500 °C. Microelectron. J..

[24] EOS PA2200 Material Data Sheet. https://www.epfl.ch/schools/sti/ateliers/wp-content/uploads/2018/05/sls_PA2200_EOS.pdf.

[25] Heat and Cold Chamber KTH-74-65/165. https://sktb-spu.ru/produkt/kamera-tepla-holoda-kth-74-65-165/.

[26] Zukri M.N.M., Al Farisi M.S., Hasegawa Y., Shikida M. (2026). Single-step laser patterning and thinning of biocompatible MEMS flow sensor. Sens. Actuators A Phys..

[27] Ohara K., Nakashima R., Takahashi H. (2024). Airflow Sensor Using Standing Laser-Induced Graphene Cantilevers. IEEE Sens. J..

[28] Ohara K., Ando R., Shimada K., Kishimoto T., Nakashima R., Takahashi H. (2025). Laser-Induced Graphene Cantilever Airflow Sensor Fabricated via Laser Cutting and Folding a Copper–Polyimide Film. Adv. Sens. Res..

